# The Role of Existential Aspects in Predicting Mental Health and Burnout

**Published:** 2018-01

**Authors:** Saeed Tabatabaei Barzoki, Parvin Rafieinia, Imanollah Bigdeli, Mahmood Najafi

**Affiliations:** 1 Semnan University, Semnan, Iran.; 2 Department of Clinical Psychology, Semnan University, Semnan, Iran.; 3 Department of Clinical Psychology, Ferdowsi University, Mashhad, Iran.

**Keywords:** *Burnout*, *Existential Aspects*, *Mental Health*

## Abstract

**Objective:** Burnout and poor mental health are among the most important issues in workplace, which may lead to serious consequences for organizations. The present study aimed at finding possible prediction of the variables based on existential aspects of responsibility, freedom, self- transcendence, and self-distance.

**Method** **:** A total of 385 junior high school and high school teachers, who worked in Tehran schools (Dist.17), participated in this cross-sectional study. They were given Maslach Burnout Inventory, General Health Questionnaire, and Existence Scale to assess burnout, mental health problems, and existence aspects. The participants were selected through one-stage cluster sampling. Data were analyzed using a stepwise multiple variable regression method.

**Results:** Responsibility, freedom, self- transcendence, and self-distance predicted nearly 38% of the variance of mental health; moreover, responsibility, freedom, and self- transcendence predicted nearly 28% of the variance of burnout altogether.

**Conclusion: **The findings of this study revealed that the existential aspects of responsibility, freedom, self-transcendence, and self-distance could be important in predicting burnout and mental health problems among teachers. However, further studies are required to investigate this possibility.

Burnout is a work-related mental health problem which has 3 dimensions: emotional exhaustion,depersonalization, and reduced personal accomplishment. Emotional exhaustion is a state of emotional reserve depletion. Depersonalization refers to one’s negative, pessimistic, and discrete attitude towards others. Reduced personal accomplishment refers to self-inefficacy and negative attitude towards oneself (1). Although burnout is not included in Diagnostic and Statistical Manual of Mental Disorders, numerous studies have revealed that it is a useful concept in describing the main features of many employees who complain about work-related chronic stress (2). The annual cost of burnout is more than $300 billion worldwide, as the world health organization has estimated that burnout will be reported as a global outbreak by the following decade (3). 

Although burnout is applicable to many employees, especially human service professions who have face-to-face encounters with clients, this term is mostly used for teachers rather than other occupational groups (4). The problems with which a teacher is encountered in the school environment may lead to experiencing burnout syndrome (5). Reviewing the scholarly literature about burnout, we found a great interest in teacher burnout. This matter has inspired the researchers to conduct further studies to identify the variables correlated with this syndrome to deeply perceive this issue (5).

Studies have corroborated the effect of burnout on teachers’ mental health (6), somatic complaints (including back pains, nausea, difficulty in breathing, dizziness, loss of appetite, and muscle tightening) (7-8), insomnia and headache (9).

However, very little attention has been paid to factors which can prevent or reduce burnout. Therefore, having knowledge about protective factors is of significant importance in facing the psychological and medical consequences of burnout (10). 

The main identified personality factors impacting teacher burnout include personality traits (11), perceived self-efficacy (12-13), existential fulfillment (14), constructive thinking (15), and work engagement (16). In the mentioned personality factors, the concept of perceived self-efficacy is compatible with constructive thinking. Evers et al. (15) argued that constructive thinking is more fundamental to an individual rather than coping strategies, as it relates to underlying patterns of thought. Accordingly, it could be stated that the existential level of an individual is more fundamental than his cognitive level because the former impacts motivations and attitudes towards life along with its possibilities and limitations. There are those who suggest that the existential construct can play a key role in teacher burnout (17). Pines (18) suggests that burnout roots in people’s need to believe that their lives and work are meaningful, and thus they, themselves, are important and meaningful. According to the existential perspective, burnout originates from experiencing meaningless feeling; it is described as a gradual development of a frustration when people strive to find meaning in their work. Langle (17) describes the genesis of burnout by referring to the concepts of existential vacuum and fulfillment. Burnout can be regarded as a special form of existential vacuum or as a deficit of fulfillment, which entails loss of interest, lack of initiative, and emotional exhaustion. 

Existential fulfillment refers to a way of life full of meaning and purpose and reveals an existential psychological approach to life (19). According to Loonstra, Brouwers and Tomic (20), existential fulfillment has a significant role in burnout factors. Langle (21) explains the genesis of burnout from an existential-analytical point of view in several stages as follows: The etiology of burnout has its origin in a non-existential attitude, which originates from a deficiency in the personal-existential fundamental motivations. The person in this situation longs for a fulfilled life but has no orientation in the most basic existential premises. Because of an illusionary sense of purpose and interpersonal skills based on motives outside of oneself, a subjective need emerges that results in a reductionistic, merely task-oriented life philosophy. This attitude lacks the deep-rooted affirmation, which guides a person to do and to be. This lost relationship, which results in emptiness, apathy, and depression, is described by Freudenberger as the final stage of the burnout cycle. For Laschinger, Wong, and Gredo (22), Shaufelli and Bakker (23), teachers who partly accept themselves and their limitations, do not lose much energy in creating situations in which they receive an amount of extra appreciation they required. Rather, they can concentrate more on what they have to do, and thus have a more chance to actualize themselves in the challenges of working with students and learning objectives. The internal drive, typical for self-actualized individuals, is related to a greater amount of work engagement, which is found to be protective against mental exhaustion. The existential conceptual framework helps the person to define his/her problems and establish a connection between burnout, family background, and sense of failure in existential desires. As mentioned by Lambie (24), the opinion that the humanistic existential tenets of congruence, self-acceptance, existential meaningfulness, personal responsibility of choice, and growth as a process are antithetical to the constructs is related to high susceptibility to burnout (eg, hopelessness, lower levels of ego development, poor self- esteem, perfectionism, and the need for approval and control). Thus, it can be inferred that a humanistic existential perspective would be an effective burnout prevention strategy. Based on this premise, the present study focused on the existential aspects that predict burnout and mental health.

## Materials and Methods

*Participants and procedure*

In this cross-sectional study, 400 junior high school and high school teachers of district 17 of Tehran were selected using one-stage cluster sampling. Then, Maslach Burnout Inventory, General Health Questionnaire, and Existence Scale were given to them. Sample size was calculated by G-power software (Version 3.1.5), considering the α of 0.05, 1-β of 0.95, and effect size of 0.33, with 4 predicator variables. Of the participants, 385 teachers with informed consent completed the questionnaires; of the participants, 58% were male and 42% were female. Inclusion criteria were as follow: at least 5 years of teaching experience, age over 25 years, and willing to participate in the study. Exclusion criteria were as follow: a non- return and failure to respond to all questions in the questionnaires.

*Research Instrument*

*Existence Scale*

This scale is a new self-rating test assessing the degree of personal fulfillment in one’s existence. The test consists of 46 items and determines the degree of existential fulfillment on the following 4 subscales: self-distance (8 items); self-transcendence (14 items); freedom (11 items); and responsibility (13) items. Internal consistency (Cronbach alpha coefficient) for the subscales was as follows: self-distance: 0.70; self-transcendence: 0.84; freedom: 0.82; responsibility: 0.83; personality: 0.87; existence: 0.90; and the total score is 0.93 (25). Maynina and Vasanov (26) performed a research and reported the test-retest reliability for each of the above subscales as follows: self-distance: 0.68; self-transcendence: 0.68; freedom: 0.66; responsibility: 0.76; personality: 0.71; existence: 0.75; and a total score of 0.75. This scale was first translated into Persian, and back-translated to make necessary revisions. The scale, then, was provided to psychology professionals to assess its face validity. After assessing face validity, the scale was implemented on 40 testees after 2 weeks to assess the test-retest reliability. The results were as follow: self-distance: 0.70; self-transcendence: 0.65; freedom: 0.64; responsibility: 0.71; and the total score was 0.68. 

*Maslach Burnout Inventory*

This questionnaire consists of 22 items, which assess 3 subscales of emotional exhaustion (9 items), depersonalization (5 items), and personal accomplishment (8 items). This questionnaire has been translated into Persian by Badri Gargari (27), with a Cronbach alpha coefficient of 0.84 for emotional exhaustion, 0.75 for depersonalization, and 0.74 for personal accomplishment. On the other hand, Akbari et al. (28) have assessed the factorial validity and psychometric properties of this inventory in Iranian EFL (English as a Foreign Language) teachers. The results demonstrated that this inventory can be used in burnout research with Persian speaking Iranian participants. In the present study, the reliability of the 3 factors of burnout (emotional exhaustion, depersonalization, and personal accomplishment) has been calculated as 0.76, 0.60, and 0.70, respectively (28).

*General Health Questionnaire*

The 28-item General Health Questionnaire was developed by Goldberg and Hillier in 1979. Using factor analysis, the items were selected from the primary 60-item form and were divided into 4 subscales: somatic symptoms, anxiety and insomnia, social dysfunction, and severe depression. Assessing the validity of the questionnaire, Yaghoubi et al. (29) have reported the cut-off point of 6 via conventional scoring method and the cut-off point of 23 via Likert scoring scale; they also demonstrated the reliability coefficient of 0.88 for the questionnaire using open-trial method. To assess reliability, 10% of the participants (90 persons) were re-evaluated one week after the first test, when a correlation of r= 0.85 was calculated and found to be significant at 99% confidence level. SCL-90-R test was given to 10% of the participants (90 persons) to assess the concurrent validity of the questionnaire. Sensitivity, specificity, and the overall misclassification rate of 90 testees in GHQ-28 were 84.2%, 94.4%, and 7.8%, respectively; however, the above rates for SCL-90-R were 80.9%, 92.7%, and 10%, respectively (30).

## Results

The average age of male participants was 34.4 (Standard deviation = 6.05), and the mean years of their teaching experience was 10.5 (Standard deviation = 5.9). The average age of female participants was 35.8 (Standard deviation = 7.1), and the mean years of their teaching experience was 12.2 (Standard deviation = 6.8). Of the participants, 60.5% were married and 39.5% were single. 

Table 1 demonstrates the descriptive statistics of the study measured variables including minimum, maximum, mean, and standard deviation. Multiple linear regression analysis was used via step-by-step method to identify the contribution of the existential subscales in determining the mental health variance. Statistical tolerance and variance inflation factor (VIF) were used to ensure that the assumption of the test (non-collinearity) was met, where the tolerance and variance inflation factor were in the ranges of 0.51 to 1 and 1 to 1.9, respectively, which indicate that the assumption of non-collinearity among predictor variables was met.

As demonstrated in Table 2, distribution of variables relating to mental health problems were as follows: responsibility: 29% (Negative coefficient of Beta indicated an inverse relationship between the scores of existential states and that of GHQ; higher scores in this questionnaire showed more severe mental health problems.); responsibility and freedom: 34%; responsibility, freedom, and self- transcendence: 37%; and responsibility, freedom, self- transcendence, and self-distance nearly 38%. Moreover, to identify the contribution of the existential subscales in determining the burnout variance, multiple linear regression analysis was used via step-by-step method. Statistical tolerance and variance inflation factor (VIF) were used to ensure that the assumption of the test (existence of no collinearity) was met, where the tolerance and variance inflation factor ranged from 0.54 to 1 and 1 to 1.8, respectively, indicating that the assumption of no collinearity among predictor variables was met.

Table 3 demonstrates the distribution of variables relating to the burnout as follows: responsibility: 22% (The negative coefficient of Beta indicated an inverse relationship between the scores of existential aspects and that of burnout.); responsibility and freedom: 27%; responsibility, freedom, and self- transcendence: nearly 28%.

**Table 1:** The Descriptive Statistics of the Study Variables

**Variable**	**Minimum**	**Maximum**	**Mean**	**Standard deviation**
Self-distance	7	63	34	6.82
Responsibility	5	78	59.34	10.86
Freedom	40	79	63.25	6.81
Self- transcendence	35	137	105.96	16.81
Burnout	0	58	18.51	11.7
General health	0	93	28.81	18.24

**Table 2:** Mental Health Regression Based on Existential Subscales

**Model**	**beta**	**R**	**R** ^2^	**Adjusted R** ^2^	***Δ*** **R** ^2^	***Δ*** **F**
Responsibility	-0.539**	0.539	0/29	0.289	0.29	156.722
Responsibility+	-0.336**	0.590	0.348	0.349	0.058	33.968
Freedom	-0.315**					
Responsibility+	-0.284**					
Freedom+	-0.274**	0.608	0.370	0.365	0.022	13.134
Self- transcendence	-0.170**					
Responsibility+	-0.314**					
Freedom+	-0.302**	0.615	0.378	0.372	0.08	5.112
self- transcendence+	-0.197**					
Self-distance	-0.115*					

R = multiple correlation coefficient; R^2 ^= Coefficient of determination; *∆*R^2 ^= the difference in the squared multiple correlations; *∆*F = the variance analysis relating to the difference in the squared multiple correlations; Beta = standard regression coefficient;

* *P* *>* *0.05*/

** *P* *>* *0.001*

**Table 3:** Burnout Regression Based on Existential Subscales

**Model**	**Beta**	**R**	**R** ^2^	**Adjusted R** ^2^	**ϪR** ^2^	**ϪF**
Responsibility	-0.474**	0.474	0.225	0.223	0.225	111.117
Responsibility+	-0.295**	0.520	0.270	0.226	0.045	23.729
Freedom	-0.278**					
Responsibility+	-0.263**					
Freedom+	-0.253**	0.528	0.279	0.273	0.08	4.431
Self- transcendence	-0.105*					

R = multiple correlation coefficient; R2 = Coefficient of determination; ∆R2 = the difference in the squared multiple correlations; ∆F = the variance analysis relating to the difference in the squared multiple correlations; Beta = standard regression coefficient /

* P> 0.05/

** P> 0.001

## Discussion

The results revealed that there was a significant relationship between the subscales of existential fulfillment (self-distance, self-transcendence, freedom, and responsibility) and teachers’ burnout and mental health. However, the relationship between self-distance and burnout was not significant because this component was one of the personality factors. Personality factors depend more on personality development than existential factors (25). Considering the relationship between the dimensions of existential fulfillment and burnout symptoms, it can be reasonably argued that teachers who accept themselves and their limitations to some extent do not lose much energy in creating situations in which they receive an amount of extra appreciation they required. Rather, they can concentrate more on the subject they have to do, and thus have a more chance to actualize themselves in the challenges of working with students and learning objectives. Internal drive, typical for self-actualized individuals, is related to a greater amount of work engagement, which is found to be protective for arising mental exhaustion (22- 23). 

The above findings are consistent with that of Langle’s (17) definition of burnout and existential significance. The present study was in agreement with Karazman (31) and Nidl (32) studies. Pines (18) argued that failure to find meaning results in burnout. He states that individual’s existential significance is evaluated by the meaning in his work. The act we do should be meaningful, useful, and important in a larger perspective. On the other hand, Langle (17) maintains that there is a significant relationship between burnout and existential fulfillment so that deficits in adopting a crucial attitude towards oneself could lead to an existential vacuum, and consequently end in a meaningless life. He states that being too much committed to work often leads to an increase risk of burnout. The balance between the optional function and being prepared to produce a flexible reaction to the demands of life through adopting a personal attitude to oneself can lay the grounds for achieving mental health and wellbeing. Wurst and Maslo (33) reported a correlation between mental health measured by TPI (Trier Personality Inventory) and existential subscales; their findings were confirmed by the present study. According to Frankl (34), lack of existential states causes the person to be influenced by the power of real world, which might be an important step towards the development of neurotic disorders. From the Peters (33) point of view, mental health would not be deteriorated by the ability to cope with the environment’s demands, and it should be recognized that humans follow the objectives they define. Langle (35) defines mental health as an individual’s ability to act in a way that has a correct meaning to him/her. He further states that humans meaningfully represent themselves not through compatibility, but though adopting a point of view towards themselves; and if the individual’s evaluation includes recognition of values in a responsible and self-motivated manner, then existential fulfillment and mental health can be attained, and this was similar to the findings of the present study.

## Limitation

The current study faces several limitations. First, this was a cross-sectional study; therefore, some considerations should be taken into account for cause-effect interpretation. Theoretical confirmation in this study implies the fact that existential fulfillment impacts burnout and mental health. However, one might find this impact in an opposite way. Causal orientations require further investigations. To resolve this limitation, a longitudinal study needs to be conducted to assess a causal relationship among existential fulfillment, burnout, and mental health. In addition to the longitudinal study, investigating a causal relationship to examine the effect of psychological intervention such as Langle’s (17) existential fulfillment attitude, using experimental designs, is also suggested. 

The second limitation is the existential fulfillment scale, which is not validated completely; nevertheless, the findings of the present study serve as the preliminary steps to validate it. Also, these findings should be replicated in other districts of Tehran, other cities and groups like workers, employees, nurses, etc. Moreover, since the research instrument in the current study was self-report, it is not clear to what extent the measurements correctly reflected the existential fulfillment, burnout, and mental health. Therefore, other evaluation methods such as interviews are suggested for further studies. 

## Conclusion

The findings of this study revealed that the existential aspects of responsibility, freedom, self-transcendence, and self-distance could be important in predicting burnout and mental health problems among teachers. However, further studies are required to investigate this possibility. This study could be a starting point in the field of intervention to determine the effectiveness of existential therapy on burnout.
